# Computational Analysis of the Molecular Mechanism of RamR Mutations Contributing to Antimicrobial Resistance in *Salmonella enterica*

**DOI:** 10.1038/s41598-017-14008-5

**Published:** 2017-10-17

**Authors:** Yen-Yi Liu, Chih-Chieh Chen

**Affiliations:** 1 Institute of Population Health Sciences, National Health Research Institutes, Miaoli, 35053 Taiwan; 2 Institute of Medical Science and Technology, National Sun Yat-sen University, Kaohsiung, 80424 Taiwan; 3 Medical Science and Technology Center, National Sun Yat-sen University, Kaohsiung, 80424 Taiwan; 4 Rapid Screening Research Center, National Sun Yat-sen University, Kaohsiung, 80424 Taiwan; 5 General Institute of Clinical Medicine, Kaohsiung Medical University, Kaohsiung, 80708 Taiwan

## Abstract

Antimicrobial resistance (AMR) in pathogenic microorganisms with multidrug resistance (MDR) constitutes a severe threat to human health. A major causative mechanism of AMR is mediated through the multidrug efflux pump (MEP). The resistance-nodulation-division superfamily (RND family) of Gram-negative bacteria is usually the major cause of MDR in clinical studies. In *Salmonella enterica*, the RND pump is translated from the *acrAB* gene, which is regulated by the activator RamA. Many MEP-caused AMR strains have high *ramA* gene expression due to mutations in RamR, which has a homodimeric structure comprising the dimerization domain and DNA-binding domain (DBD). Three mutations on the dimerization domain, namely Y59H, M84I, and E160D, are far from the DBD; the molecular mechanism through which they influence RamR’s binding affinity to the *ramA* gene promoter and consequently disrupt RamA remains unclear. The present study conducted molecular dynamics simulations, binding free energy calculations, and normal mode analysis to investigate the mechanism through which Y59H, M84I, and E160D mutations on the dimerization domain influence the binding affinity of RamR to the *ramA* promoter. The present results suggest that the three mutations alter the RamR structure, resulting in decreased DNA-binding affinity.

## Introduction

In recent years, antimicrobial resistance (AMR) has become a global concern. Pathogens with AMR can threaten human life; if pathogens with AMR infect inpatients, their treatment periods tend to be prolonged, thereby increasing healthcare costs. The abuses of antimicrobial drugs in clinics and agricultural settings imperceptibly culture many AMR strains in nature. For example, carbapenem-resistant *Klebsiella pneumonia*^1^ and colistin-resistant *Enterobacteriaceae*^2^ can pose severe threats to human life.

The mechanism of AMR includes target alteration, drug inactivation, decreased permeability, and increased efflux^3^. Multidrug resistance (MDR) is an important aspect of AMR, and the multidrug efflux pump (MEP)^4^ is one of the essential mechanisms that mediate MDR. Several MEP-caused MDR pathogens include *Salmonella enterica*^5^, *Escherichia coli*^6^, *Staphylococcus aureus*^7^, and *K*. *pneumonia*^8^. The bacterial MEP can be classified into five families^9^: resistance-nodulation-division superfamily (RND family)^10^, major facilitator superfamily^11^, ATP-binding cassette superfamily^12^, small MDR family^13^, and multidrug and toxic compound extrusion family^14^. Of the five MEP families, the RND family is the major cause of MDR in clinical studies^15^; for instance, the *acrAB* gene in *E*. *coli* and *S*. *enterica*^16^. Several studies on RND family-caused bacterial MDR^17,18^ have reported that RamA, the activator of the *acrAB* gene, is an essential target for elucidating the MDR mechanism, particularly in *S*. *enterica*^19^ and *K*. *pneumonia*^20,21^. In 2008, Abouzeed *et al*.^22^ identified *ramR*, a TetR family of transcriptional repressor gene, which is located upstream of the *ramA* gene. RamR binds to the promoter region of the *ramA* gene to repress *ramA* gene transcription, thereby resulting in MDR. Several studies have reported different RamR mutations in the MDR strains of *S*. *enterica* and *K*. *pneumonia* that can reduce the repression of the *ramA* gene^22–24^. However, the molecular mechanism through which mutations, such as Y59H, M84I, and E160D^22,25,26^, on the dimerization domain of RamR reduce its ability to repress *ramA* gene expression remains unclear. The present study conducted molecular dynamics (MD) simulations and binding free energy calculations to investigate the mechanism through which Y59H, M84I, and E160D mutations in the dimerization domain cause MDR by assessing the binding events between the RamR protein and the *ramA* promoter.

We constructed four structures, specifically, the wild-type RamR (WT-RamR) and three mutants (MTs), namely Y59H-RamR, M84I-RamR, and E160D-RamR. The root mean square deviation (RMSD) and RMS fluctuation (RMSF) simulation results suggested that mutations in the dimerization domain of RamR might stabilize the overall structure but make structural changes in the DNA-binding domain (DBD), which could influence RamR’s binding affinity to the *ramA* promoter DNA. To further investigate the mutation-induced changes in interaction properties, the total free energy of the MT and WT complexes was calculated to obtain the binding free energy. In addition, normal mode analysis (NMA) was performed to determine whether the mutations alter the dynamics networks to influence large-scale domain motion. The binding free energy calculations and NMA results were in accordance with the observations from MD simulations.

## Materials and Methods

### Three-dimensional models of MT-RamR structures

We selected 3VVX^27^ as the template to run MODELLER (9v8)^28^ for predicting the structures of Y59H-, M84I-, and E160D-RamR. Three mutant RamR models were constructed on the basis of the sequence and structural alignment of the 3VVX template. The qualities of all modeled structures were assessed through Ramachandran plots^29^. The steepest descent algorithm^30^ was used for structural optimization (energy minimization), and PyMOL^31^ was used for all three-dimensional (3D) structures and charge distribution presentation.

### Model of the RamR–DNA complex structure

Because the structure of the RamR**–**DNA complex is yet to be solved, we predicted its structure. Previous studies have demonstrated that RamR acts as a repressor and binds to the promoter region (TATAATGAGTGCTTACTCACTCATAATC) of the *ramA* gene to inhibit *ramA* gene expression^32^. Therefore, we used 3D-DART^33^ to construct a 3D model of the ramA promoter. Furthermore, we used HADDOCK (v2.2)^34,35^ to perform the docking of the promoter DNA molecule to the RamR protein for constructing the RamR–DNA complex structural model.

### Molecular dynamics simulation

Molecular dynamics (MD) simulations were performed using GROMACS software (ver. 5.1.4)^36^. The Amber99 force field was used for energy calculations. The models were solvated with TIP4P water molecules and simulated in a cubic box with periodic boundary conditions. The energy of the models was first minimized using the steepest descent algorithm. The simulations were performed in the canonical NVT (constant number of particles, volume, and temperature) and NPT (constant number of particles, pressure, and temperature) ensembles through the position-restrained MD simulation for 100 ps, respectively. The linear constraint solver algorithm was used to constrain the bonds^37^. The MD simulations were performed at constant pressure and temperature for 20 ns using an integration time step of 2 fs. The nonbonded interactions were cutoff at 10 Å. The coordinates from the MD simulations were saved at every 100 time steps.

### Trajectory analysis

To understand the structural and functional implications of the amino acid substitutions in the dimerization domain on the DBD, the trajectories of the WT and MT structures were analyzed for several structural properties as a function of time: (A) The RMSD of the Cα atoms of the DBD with respect to the starting conformation, *r*^*ref*^, was calculated as $RMSD(t)={[\frac{1}{M}{\sum }_{i=1}^{N}{m}_{i}{|{r}_{i}(t)-{r}_{i}^{ref}|}^{2}]}^{1/2}$ where $M={\sum }_{i}{m}_{i}$ and ${r}_{i}(t)$ is the position of atom *i* at time *t* after last square fitting of the structure to the reference structure; (B) the RMSF of the backbone Cα atoms with respect to the starting conformation was calculated as follows. The RMSF is a measure of the deviation between the position of particle *i* and some reference position: $RMS{F}_{i}={[\frac{1}{T}{{\sum }_{{t}_{j}=1}^{T}|{r}_{i}({t}_{j})-{r}_{i}^{ref}|}^{2}]}^{1/2}$ where *T* is the time over which one wants to average and ${r}_{i}^{ref}$ is the reference position of particle *i*. Typically, this reference position will be the time-averaged position of the same particle, that is, ${r}_{i}^{ref}={\overline{r}}_{i}$; (C) the Debye–Waller factor (B-factor), which reflects the fluctuation of an atom about its average positions; each Cα atom was calculated from the last 5 ns of the MD trajectories. The B-factor was calculated from the average RMSF per residue by using the relation $B-factor=\frac{8}{3}\times {\pi }^{2}\times RMS{F}^{2}$. A large B-factor indicates a high flexibility of the atom.

### Binding free energy calculation

The pmx tool was used to construct the hybrid topologies of the system used in the free energy calculations^38,39^. This tool automatically generates hybrid structures and topologies for amino acid mutations that represent the two physical states of the system. After obtaining this hybrid structure, free energy simulations were performed using GROMACS. From the 10-ns equilibrated trajectories, 100 snapshots were extracted, and a rapid 100-ps simulation was performed starting from each frame. The lambda (λ) ranged from 0 to 1 and from 1 to 0 for the forward and backward integrations, respectively, thus describing the interconversion of the WT to MT systems and the MT to WT systems, respectively. Finally, the pmx tool was used to integrate over the multiple curves and subsequently estimate free energy differences using the fast-growth thermodynamic integration approach, which relies on the Crooks–Gaussian Intersection (CGI) protocol^40^. The ΔG values for the mutations in the DNA-free and DNA-bound structures were calculated to construct a double free energy difference (ΔΔG). The final ΔΔG values indicate whether mutations stabilize or destabilize the protein in the DNA-bound state.

### Normal mode analysis

Normal mode analysis (NMA) is commonly adopted to estimate dynamics based on structures^41–44^. A low-frequency large-scale motion usually reflects real-world biological functional motions^41,45^. Although NMA is powerful, it is very time-consuming. In this study, we used Elastic Network Model (ENM)^46–48^, a coarse-grained NMA method, to compute the low-frequency motions of the RamR protein using the web-service DynOmics^49^. In addition, pair-wise residue correlation maps were constructed.

## Results

### Predicted MT-RamR structures

**Figure 1:**
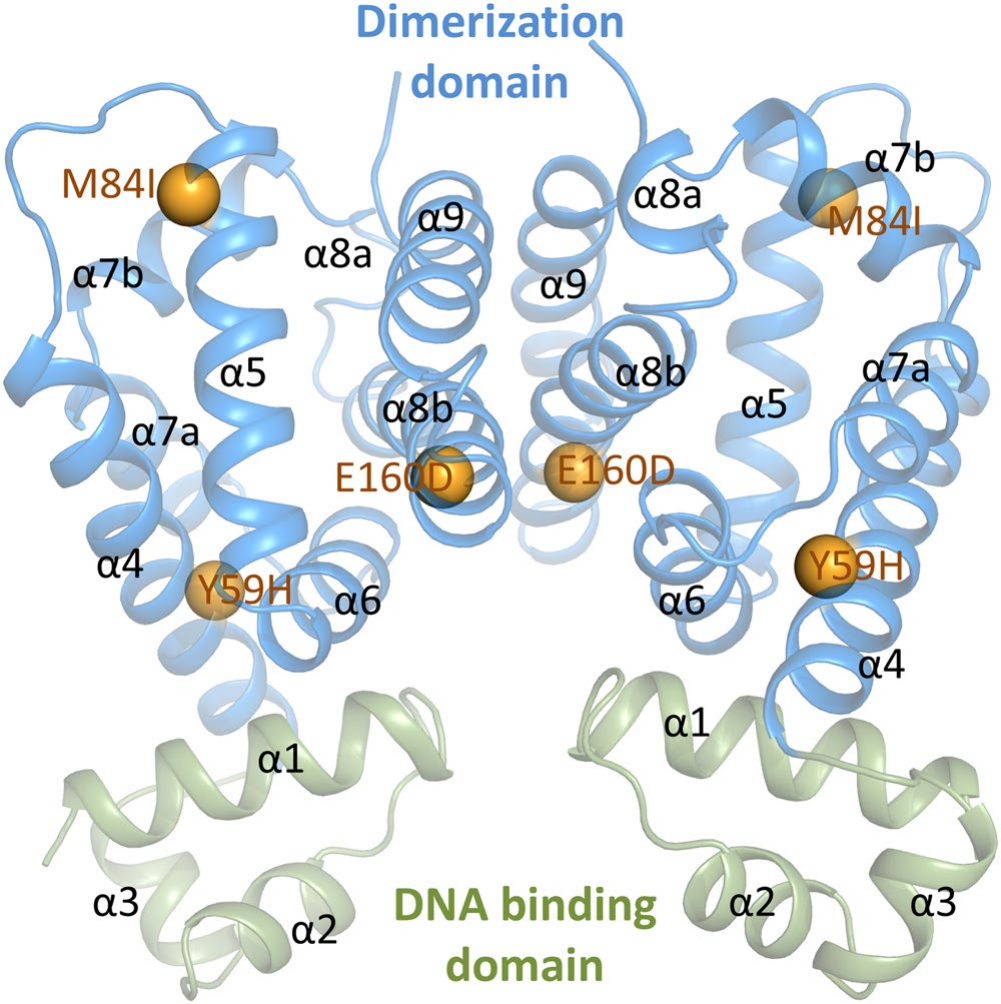
Protein structure of the human RamR dimer. The structure contains the DBD (green) and the dimerization domain (blue). The RamR mutations of Y59H (α4), M84I (α5), and E160D (α8b) are highlighted in orange spheres. The structural representation is generated by PyMOL.

The MT-RamR models were constructed using the published RamR crystal structure (Protein Data Bank Id: 3VVX) from *S*. *typhimurium* as the template; it is an all-alpha helix structure consisting of two dimerization domains and two DBDs (Fig. 1). The substitution residues, namely Y59, M84, and E160, are all located at the dimerization domain. Y59 is located at the boundary of the dimerization domain and the DBD; M84 is at the top of the dimerization domain; and E160 is at the dimerization surface between the two monomers. Figure 1 shows all amino acid substitutions in the RamR structure. One of the main purposes of the present study was to thoroughly examine the structural flexibility of the DBD with respect to the amino acid substitutions on the dimerization domain.

### Predicted RamR–DNA complex structures

**Figure 2:**
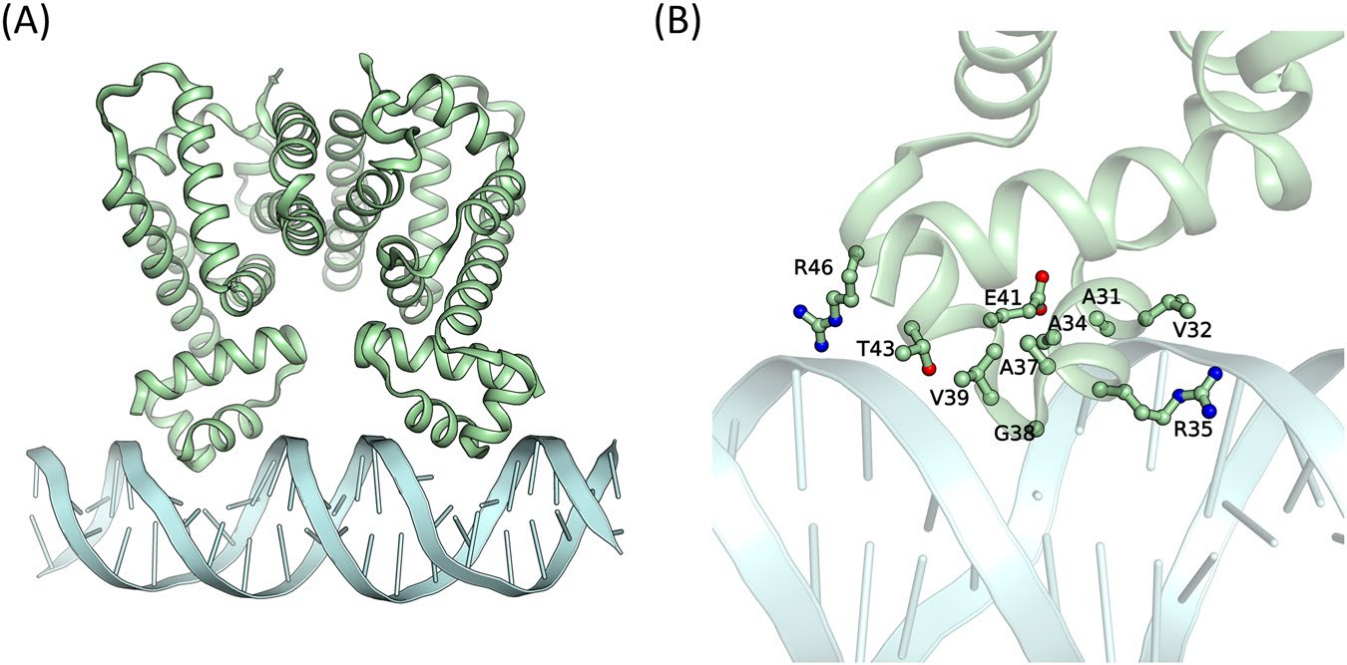
Ribbon representation of the RamR–DNA complex model structure. (**A**) The structure of the RamR (green)–DNA (cyans) complex, obtained through HADDOCK prediction. (**B**) A close view of the human RamR binding residues with DNA.

To construct the RamR–DNA complex structures, we first used 3D-DART^33^ to build the 3D coordinates of the RamR binding promoter (TATAATGAGTGCTTACTCACTCATAATC), followed by the HADDOCK^34,35^ service tool. Figure 2A presents the predicted complex model. On the basis of the study of Yamasaki *et al*.^27^, the fragment from residues 2 to 42 of RamR was defined as the DBD region. In our predicted complex structure, residues A31, V32, A34, R35, A37, G38, V39, E41, T43, and R46 in both chain A and chain B were involved in DNA-binding. Figure 2B shows a close view of the DNA-binding surface.

### RMSD analysis of the DBDs of the WT- and MT-RamR structures

**Figure 3:**
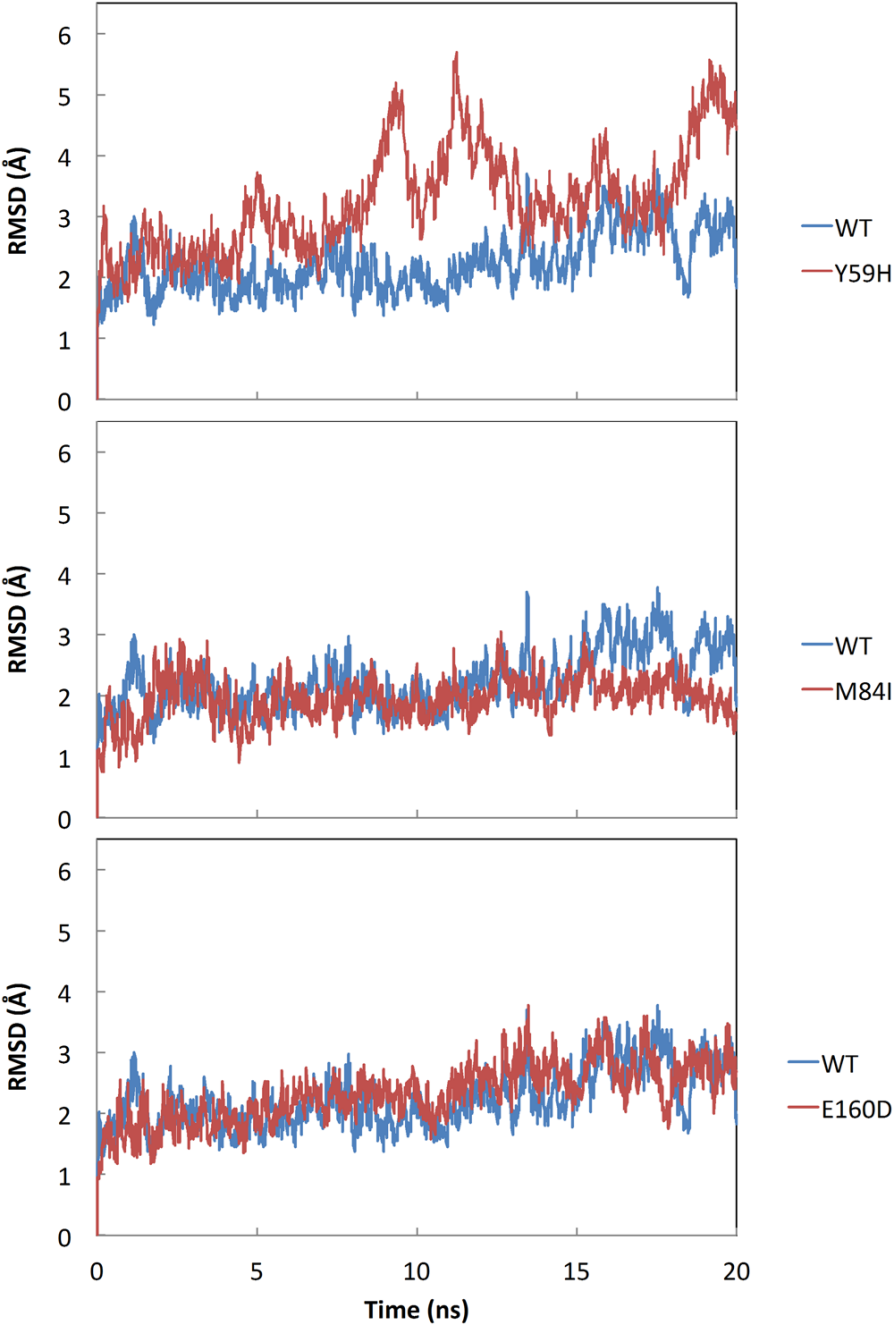
RMSD plot of the DBD. Comparison of the RMSD plots of the DBDs of WT and MT (**A**) Y59H, (**B**) M84I, and (**C**) E160D RamR structures with respect to the initial conformation during the course of the simulation. WT and MT plots are presented in blue and red, respectively.

The trajectories from MD simulations of the WT- and MT-RamR–DBDs in the explicit solvent were calculated. The RMSD values of the Cα atoms for WT- and MT-RamR–DBDs with regard to the initial structures were plotted (Fig. 3). The quality and convergence of the simulated dynamics trajectories can be estimated by assessing the RMSD values. For the simulations of the WT- and MT-RamR–DBDs, the obtained data demonstrated that after a rapid increase during the first 0.2 ns, the trajectories were stable with the average values of 2.22, 1.96, and 2.32 Å for WT-, M84I-, and E160D-RamR–DBDs, respectively, except for Y59H-RamR–DBD. The RMSD profiles of the DBDs with respect to the initial conformation of WT and MT structures during the course of the simulations were calculated. The RMSD values showed a large increase as a function of time for the Y59H-RamR–DBD compared with the WT-RamR–DBD. For the M84I- and E160D-RamR–DBDs, the RMSD variations were nearly similar to those of the WT-RamR–DBD (Fig. 3).

### Residue fluctuation of the WT and MT RamR structures

**Figure 4:**
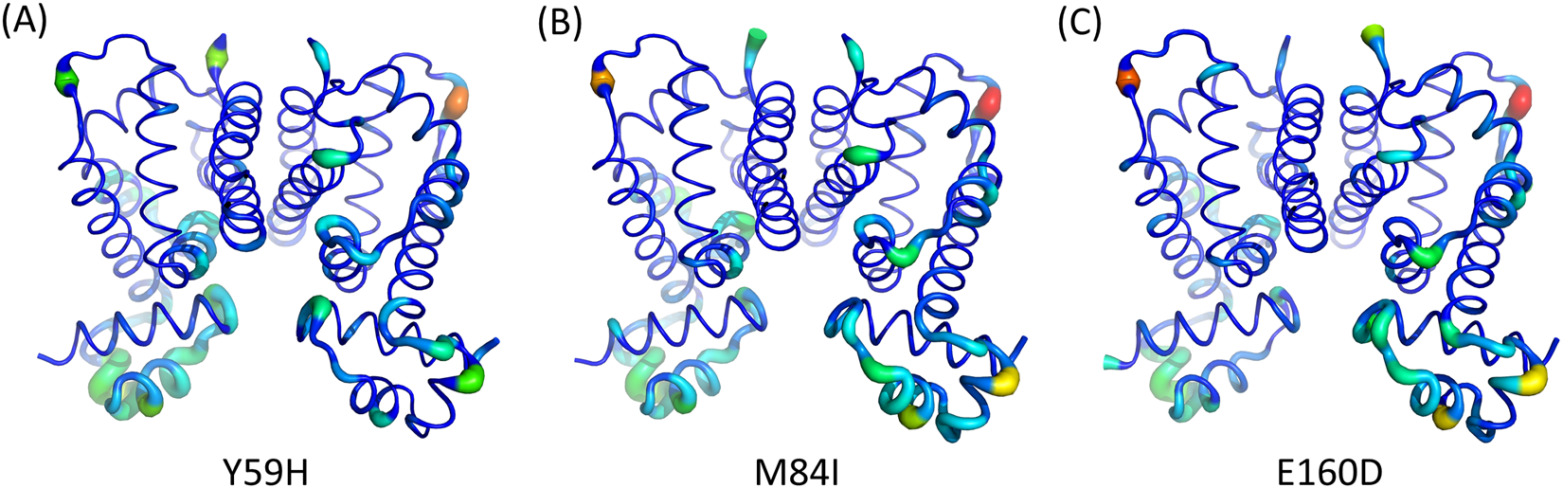
Analysis of the atomic fluctuations. The structures of (**A**) Y59H, (**B**) M84I, and (**C**) E160D RamR proteins are created as cartoon putty representations; blue and red represent the lowest and highest differences, respectively, of B-factor values compared with WT-RamR. In addition, the size of the tube reflects the differences in B-factor values; the larger the df-B values, the thicker the tube.

The overall trend of the RMSF profiles of Y59H-, M84I-, and E160D-RamR was similar to that of WT-RamR, except for their DBDs, which exhibited different values than that of WT-RamR (Figure S1). We calculated the differences in RMSF values between MT- and WT-RamR structures for each Cα atom (B-factor differences, df-B), followed by the mapping of the absolute values of df-B to the RamR structures (Fig. 4) for clear investigation of the fluctuation differences. The B-factor analysis demonstrated that the major fluctuation differences were at the DBD, comprising most of the residues relevant to the DNA-binding functionality predicted in our RamR–DNA complex model, such as V32, R35, T43, and R46, for all three MT-RamR structures (Fig. 4).

### RamR–DNA binding free energy

**Figure 5:**
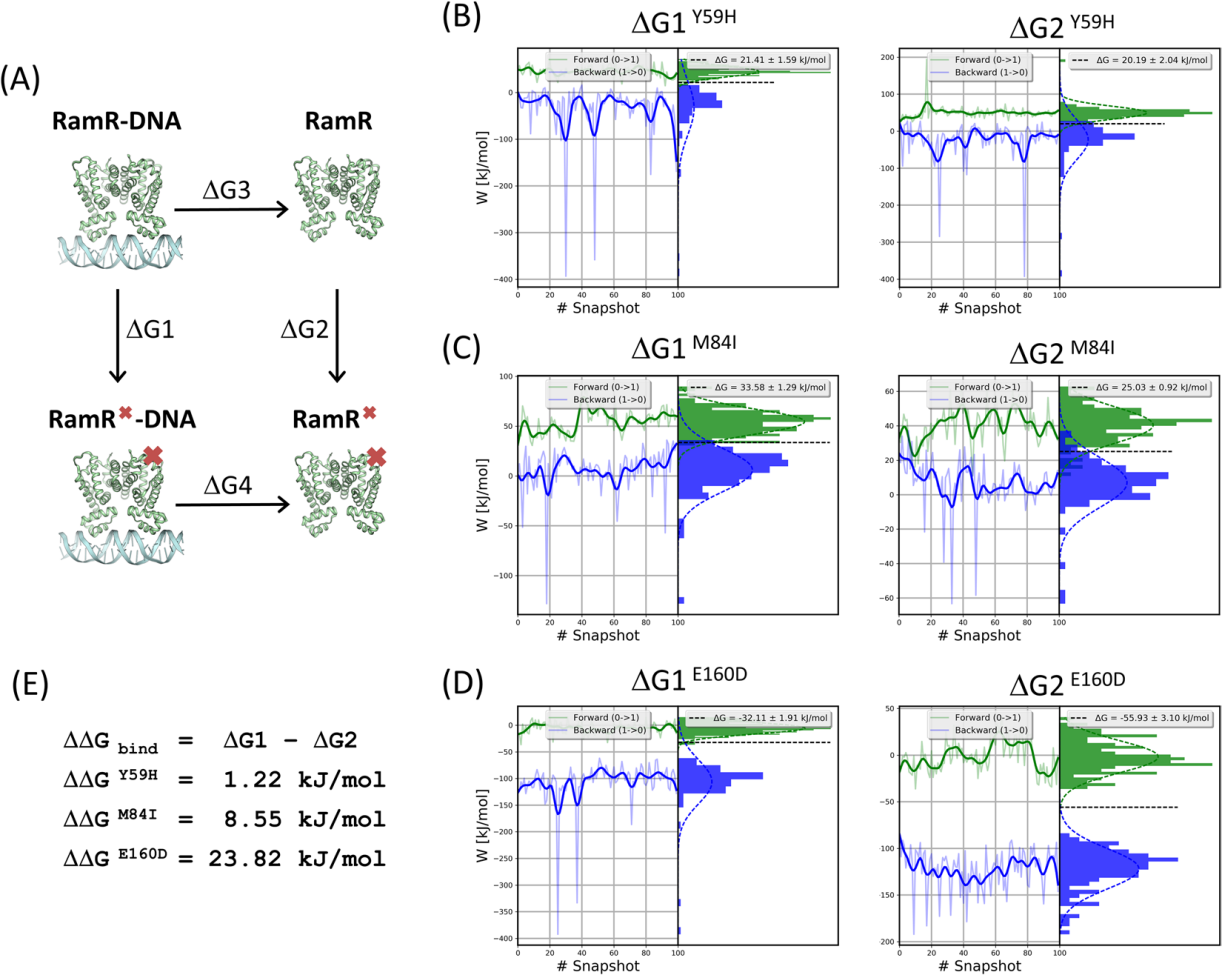
Analysis of the RamR-DNA binding free energy. (**A**) Thermodynamic cycle for binding affinity calculations. Distribution of the work values over time for an alchemical free energy simulation of (**B**) Y59H, (**C**) M84I, and (**D**) E160D mutations for the DNA-free (left panel) and DNA-bound (right panel) RamR proteins. The left plot in each result shows the work values obtained from the integration of $\delta {H}_{\lambda }/\delta \lambda$ as a function of the sampling time of the equilibrium states. The right plot in each result shows the histograms of work values from which the free energy was calculated. (**E**) In addition, the binding free energies caused by mutations (${\rm{\Delta }}{\rm{\Delta }}{G}_{bind}$) were calculated.

The RamR–DNA binding free energy differences between WT and MT structures were calculated in a series of alchemical free energy simulations and using the CGI protocol^40^. The DNA-binding affinity differences (ΔΔ*G*_bind_) were calculated according to Δ*G*_1_ (DNA-bound)–Δ*G*_2_ (DNA-free), as shown in the thermodynamic cycle (Fig. 5A). Notably, stabilizing mutations have negative ΔΔ*G* values. We adopted the CGI protocol to calculate the binding affinity differences between DNA and RamR and the single point mutations. For DNA-free and DNA-bound structures, the free energy differences for Y59H were 20.19 ± 2.04 and 21.41 ± 1.59 kJ/mol (Fig. 5B), for M84I were 25.03 ± 0.92 and 33.58 ± 1.29 kJ/mol (Fig. 5C), and for E160D were −55.93 ± 3.10 and −32.11 ± 1.91 kJ/mol (Fig. 5D), respectively. The resulting binding free energy differences (ΔΔ*G*_bind_) between WT- and Y59H-, M84I-, and E160D-RamR were 1.22, 8.55, and 23.82 kJ/mol, respectively (Fig. 5E). The DNA-binding affinity was disrupted in all mutants (ΔΔ*G*_bind_ > 0).

### Normal mode analysis

**Figure 6:**
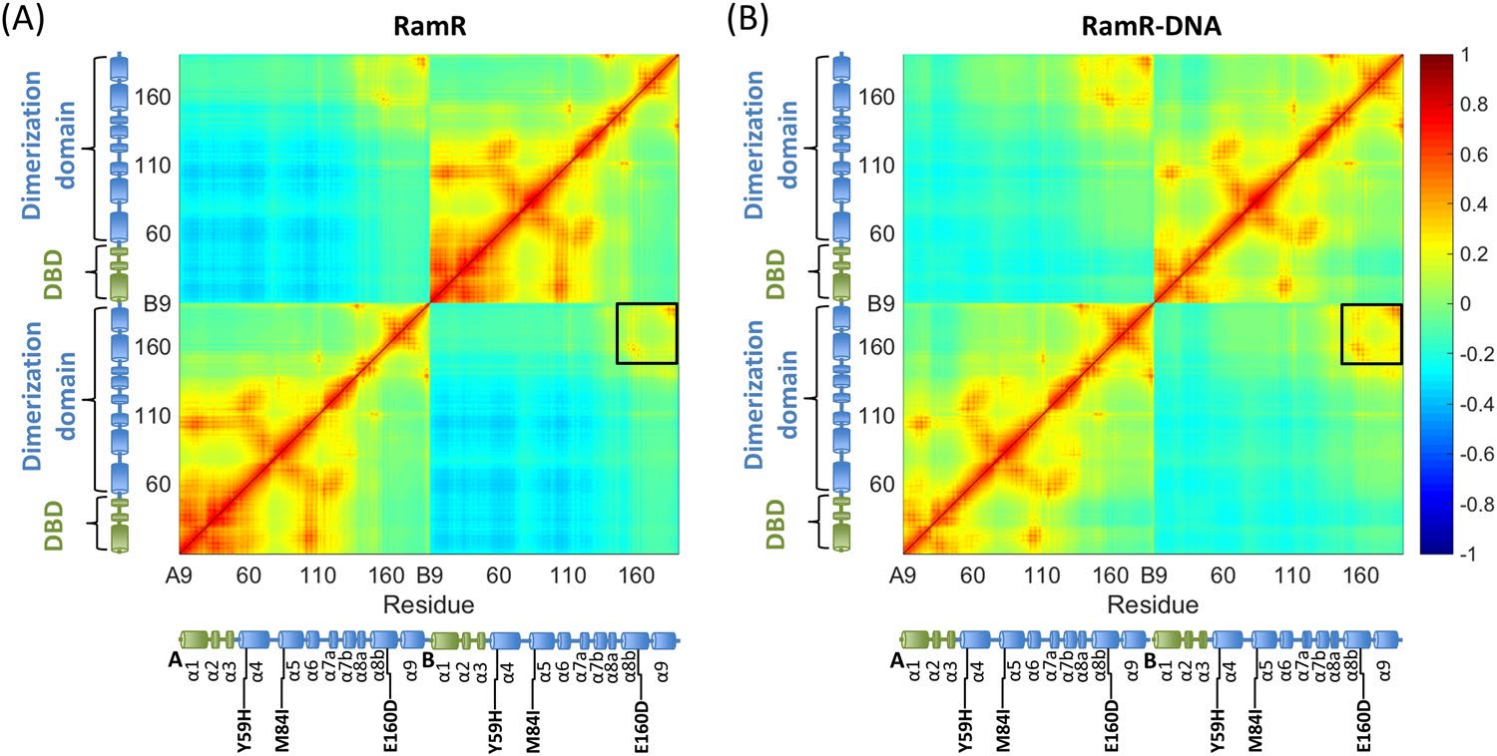
Cross-correlation map obtained from the ENM for the (**A**) DNA-free and (**B**) DNA-bound RamR proteins. A negative value refers to an anticorrelation between residue fluctuations (blue), whereas a positive value indicates concerted motion in the same direction (red).

Because normal mode analysis (NMA) is very time-consuming, we used the coarse-grained ENM^46–48^. The normal modes of WT-RamR and WT-RamR–DNA structures were calculated. For investigating the motional correlations between residues, we combined the 10 slowest modes to calculate the correlation heat maps for WT-RamR (Fig. 6A) and WT-RamR–DNA (Fig. 6B). The covariance matrix between residues represented by their Cα atoms was obtained from the ENM. The two covariance matrices indicated that the N-terminal fragment (α1–α7) differed markedly from the C-terminal fragment (α8, α9) and showed increased levels of motional correlation (red, self-chain) and anticorrelation (blue, opposite chain) in the DNA-free RamR protein. This finding is consistent with the fluctuations calculated for the DNA-free RamR protein (Figure S1), which demonstrated that the amplitude of the fluctuations is larger than that in the RamR–DNA complex (Figure S2), particularly in the DNA-binding fragment. Compared with the DNA-free RamR protein, the RamR–DNA complex exhibited increased levels of motional correlation either in the self-chains or opposite chains of the dimerization fragment (α8, α9).

## Discussion

The present study reveals that Y59H, M84I, and E160D mutations in the dimerization domain of RamR, which have been reported as MDR-relevant substitutions^22,25,26^, might affect RamR’s binding affinity to the *ramA* promoter by altering the dynamics properties of the DBD. Previous study have suggested that amino acid residues Y59, M84, and E160 are considered to be involved in RamR substrate recognition and amino acid substitutions at these positions might reduce the DNA-binding affinity according to expand the distance between the N-terminal helix-turn-helix motifs in the DBD^27^. This inference could be also observed in our dynamics and binding free energy analysis, our studies demonstrate that these mutations could disrupt the RamR–DNA binding affinity through changing the dynamics networks of the RamR structure (Fig. 5).

The stability analysis and RMSD profiles reveal that the simulated Y59H-RamR structure exhibits obvious instability compared with the WT-RamR structure (Fig. 3). The Y59 residue is located at the junction of the DBD and the dimerization domain; therefore, a mutation at this position might affect the relevant collective motions relevant to DNA-binding functionality. The highly correlated motions of residue 59 and the DBD region (residues 2–42) can be observed in the correlation heat map (Fig. 6) that clearly demonstrate the influence of residue 59 mutated. The RMSF profile of Y59H-RamR shows that the fluctuations of the DBD region have obvious changes compared with WT-RamR (Figure S1). We mapped the fluctuation differences on the RamR structure and observed that the DBD region has obvious dynamics changes after the substitution of histidine for tyrosine at residue 59 (Fig. 4). The binding free energy calculations reveal that the Y59H mutation may slightly disrupt the binding affinity of RamR to DNA (Fig. 5).

The RMSD profile of M84I-RamR revealed no influence on the structural stability compared with WT-RamR (Fig. 3). The RMSF profile of M84I-RamR show clear fluctuation changes in the DBD compared with that of WT-RamR (Figure S1). To visualize more clearly, the RMSF differences between WT- and M84I-RamR were mapped to the M84I RamR structure to observe the more fluctuation changes occurring at the DBD region (Fig. 4). NMA reveal that because of the shear motion of the two domains, residue M84 forms a hinge (Fig. 6). The binding free energy calculations demonstrate that the M84I mutation may destabilize the RamR structure then disrupt the binding affinity of RamR to DNA (Fig. 5).

Residue 160 is located at the interface between the two monomers (Fig. 1). The cross-correlation maps (Fig. 6) indicate that the positive correlated motions are found in these regions (black squares) either in DNA-free or DNA-bound states of RamR. Therefore, mutation occurring at this position might strongly influence the dynamic networks of the RamR structure. However, meaningful differences were not observed between the RMSD, and RMSF profiles, possibly because the side chains are similar between the WT and the mutant (from glutamic acid to aspartic acid). The mutation free energy calculations also reveal that the E160D mutation may stabilize the RamR protein both in DNA-free and DNA-bound states (Δ*G* < 0) (Fig. 5D) but this mutation does not contribute to increase the binding affinity of RamR to DNA (ΔΔ*G*_bind_ > 0) (Fig. 5E). The E160D mutated RamR has a tendency to the DNA-free form.

In addition, we also performed a simulation to analyze the RamR–RamR duplex dimerization free-energy changes upon these three mutations (Figure S3). For RamR monomer and dimer structures, the free energy differences for Y59H were 19.49 ± 1.15 and 16.46 ± 0.81 kJ/mol, for M84I were 14.29 ± 0.86 and 11.96 ± 0.91 kJ/mol, and for E160D were −36.78 ± 0.92 and −14.24 ± 1.05 kJ/mol, respectively (Figure S3). The resulting dimerization free-energy differences (ΔΔ*G*_dimer_) between WT- and Y59H-, M84I-, and E160D-RamR were −3.03, −2.33, and 22.54 kJ/mol, respectively (Figure S3). Compared with WT RamR, two mutants (Y59H and M84I) showed a slightly lower dimerization free energy for RamR dimer, suggesting that these mutations slightly increased the dimerization affinity of RmaR dimer (Figure S3), but these mutations do not contribute to increase the binding affinity of RamR to DNA (ΔΔ*G*_bind_ > 0) (Fig. 5). By contrast, mutants E160D showed a higher dimerization free energy, suggesting the decreased dimerization affinity of RmaR dimer. Interestingly, the position of E160D is just located at the binding interface of the RamR dimer. It is reasonable to assume that the reduction of the RamR-DNA binding affinity on E160D mutant is mainly caused by its instability in RamR duplex form.

## Conclusion

In this study, we have applied computational methods to understand the structural implications of Y59H, M84I, and E160D in RamR that are related to induce bacterial drug resistance. Our findings underscore that the Y59H, M84I, and E160D mutations may exhibit different behavior as compared to RamR WT, especially in DNA binding domain. In summary, our simulated results can properly in accordance with current study results, which demonstrated that amino acid substitutions of Y59H, M84I, and E160D in RamR might reduce its DNA-binding affinity.

## Electronic supplementary material

Supplementary figures

## Data Availability

All data generated or analysed during this study are included in this published article (and its Supplementary Information files).
