# Author Correction: Computational Analysis of the Molecular Mechanism of RamR Mutations Contributing to Antimicrobial Resistance in *Salmonella enterica*

**DOI:** 10.1038/s41598-018-32185-9

**Published:** 2018-09-18

**Authors:** Yen-Yi Liu, Chih-Chieh Chen

**Affiliations:** 1 Institute of Population Health Sciences, National Health Research Institutes, Miaoli, 35053 Taiwan; 2 Institute of Medical Science and Technology, National Sun Yat-sen University, Kaohsiung, 80424 Taiwan; 3 Medical Science and Technology Center, National Sun Yat-sen University, Kaohsiung, 80424 Taiwan; 4 Rapid Screening Research Center, National Sun Yat-sen University, Kaohsiung, 80424 Taiwan; 5 General Institute of Clinical Medicine, Kaohsiung Medical University, Kaohsiung, 80708 Taiwan

Correction to: *Scientific Reports* 10.1038/s41598-017-14008-5, published online 17 October 2017

In the original version of this Article, there was a repeated error in which “*acrAB*” was incorrectly written as “*arcAB*”.

The original version of this Article also contained an error in the Materials and Methods.

“The steepest descent algorithm^30^ was used for structural optimization (energy minimization), and PyMO^31^ was used for all three-dimensional (3D) structures and charge distribution presentation.”

now reads:

“The steepest descent algorithm^30^ was used for structural optimization (energy minimization), and PyMOL^31^ was used for all three-dimensional (3D) structures and charge distribution presentation.”

These errors have now been corrected in the HTML and PDF versions of this Article.

