# Supplementary material for: Computational Analysis of the Molecular Mechanism of RamR Mutations Contributing to Antimicrobial Resistance in Salmonella enterica
Source: Sci Rep. 2017 Oct 17;7:13418. doi: 10.1038/s41598-017-14008-5 (PMC5645378; doi:10.1038/s41598-017-14008-5)
Supplement: Supplementary file 1 — Supplementary figures [file 41598_2017_14008_MOESM1_ESM.pdf]

Computational Analysis of the Molecular Mechanism of RamR Mutations Contributing to Antimicrobial Resistance in *Salmonella enterica*

Yen-Yi Liu [1] & Chih-Chieh Chen [2,3,4,5]*

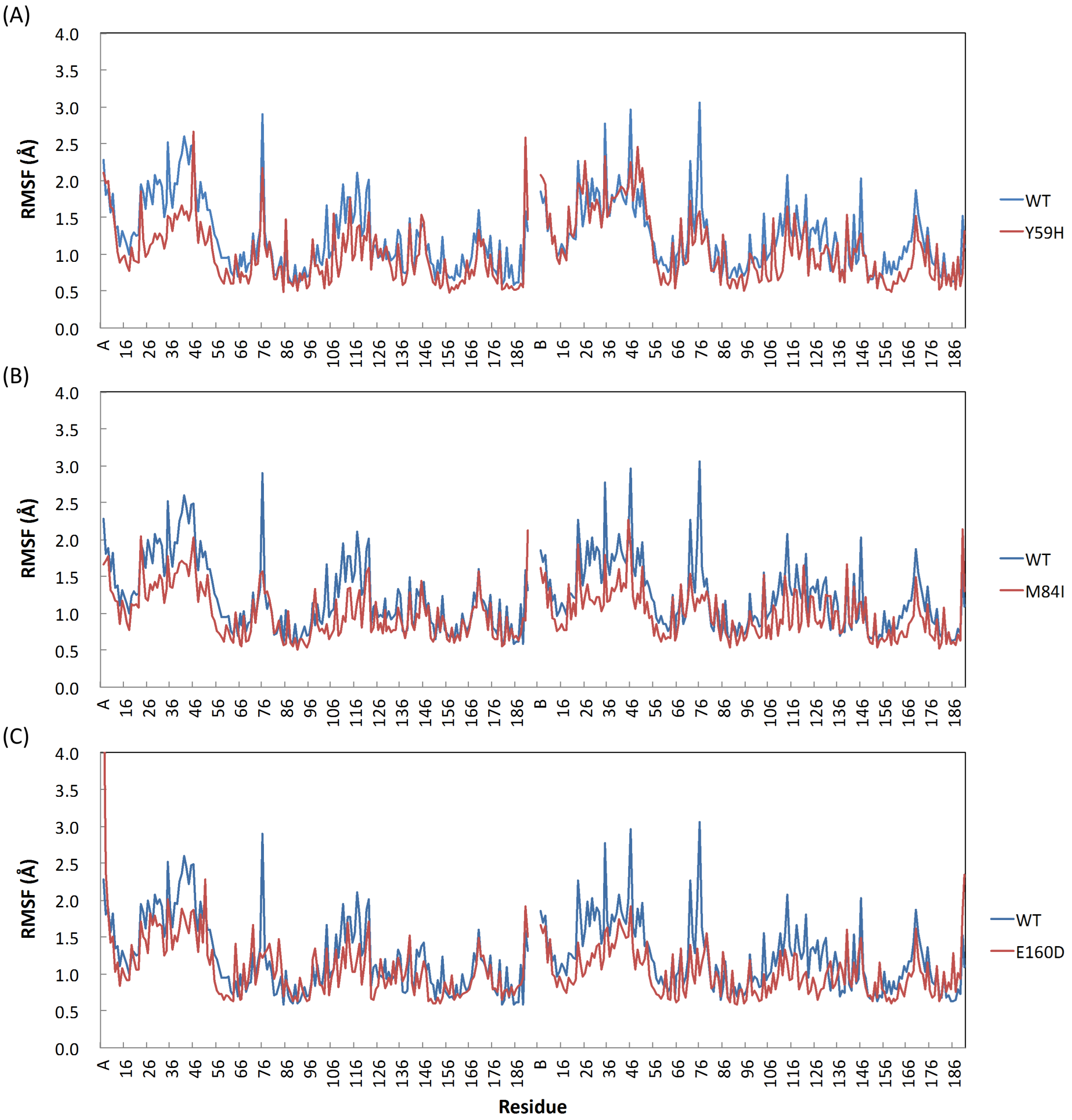

Figure S1. Analysis of the atomic fluctuations in the DNA-free (A) Y59H-, (B) M84I-, and (C) E160D-RamR proteins.

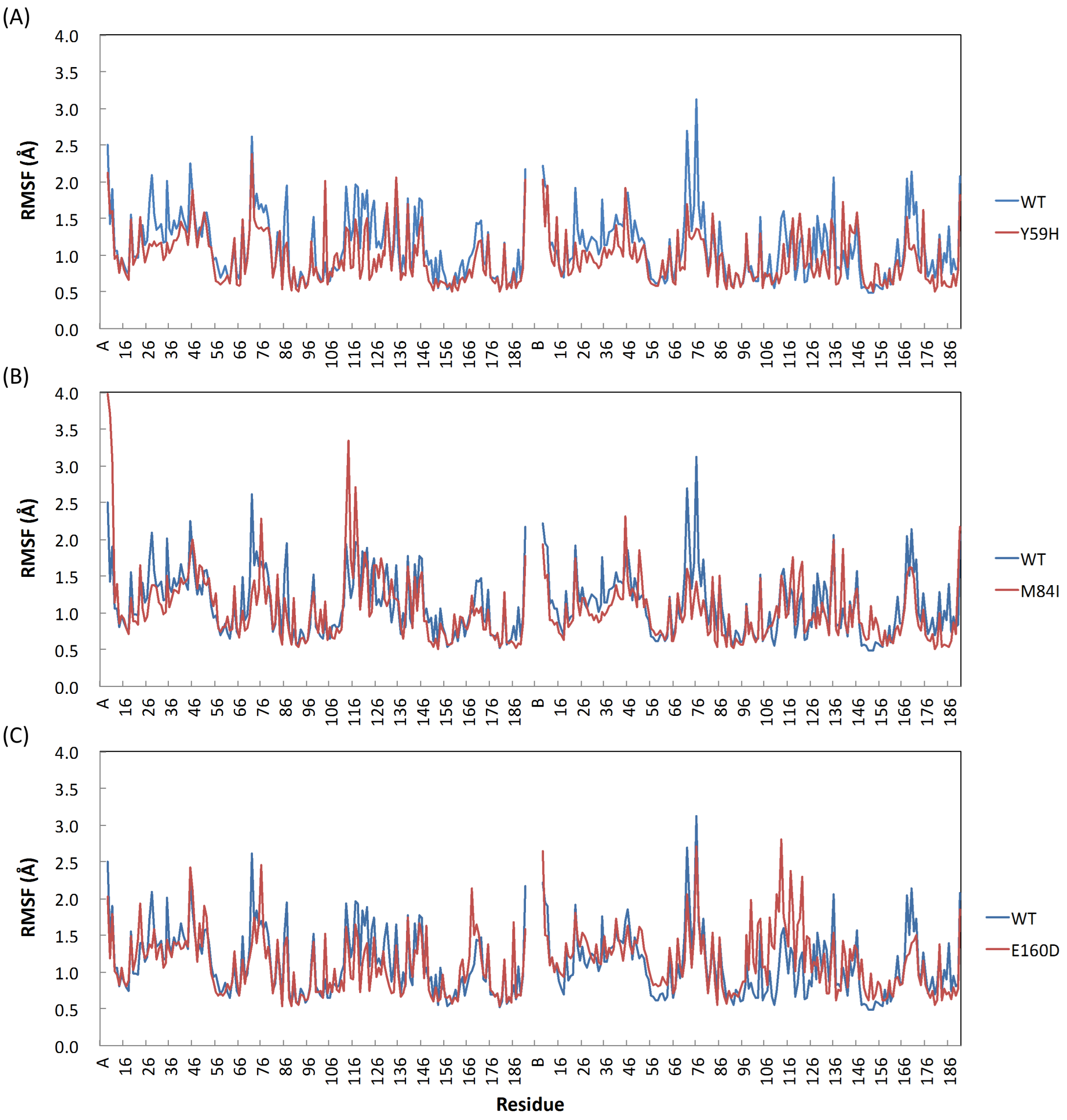

Figure S2. Analysis of the atomic fluctuations in the DNA-bound (A) Y59H-, (B) M84I-, and (C) E160D-RamR proteins.

(A)

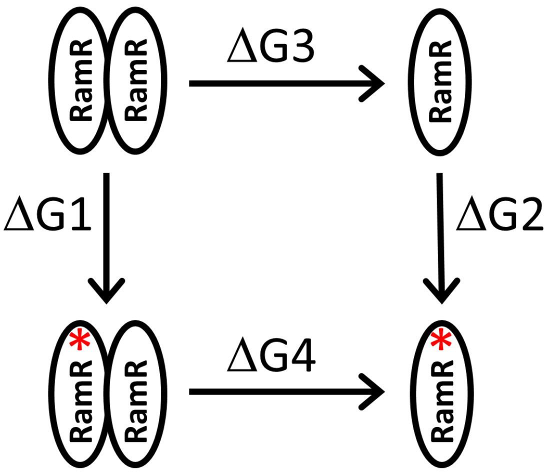

(B)

$$\Delta\Delta G_{dimer} = \Delta G1 - \Delta G2$$

$$\Delta\Delta G^{Y59H} = 16.46 - 19.49 = -3.03 \text{ kJ/mol}$$

$$\Delta\Delta G^{M84I} = 11.96 - 14.29 = -2.33 \text{ kJ/mol}$$

$$\Delta\Delta G^{E160D} = -14.24 - (-36.78) = 22.54 \text{ kJ/mol}$$

Figure S3. Analysis of RamR–RamR duplex dimerization free-energy changes upon mutations. (A) The thermodynamic cycle used for the analysis of binding free energies for the dimerization of RamR dimer. The red stars indicate the MT-RamR. (B) The RamR dimerization free-energy changes ($\Delta\Delta G_{dimer}$) upon on mutations Y59H, M84I, and E160D.
